# GPs’ decisions about prescribing end-of-life anticipatory medications: a qualitative study

**DOI:** 10.3399/bjgp20X712625

**Published:** 2020-09-08

**Authors:** Ben Bowers, Sam S Barclay, Kristian Pollock, Stephen Barclay

**Affiliations:** Queen’s Nurse, NIHR School for Primary Care Research PhD student;; Primary Care Unit, Department of Public Health and Primary Care, University of Cambridge, Cambridge.; Nottingham Centre for the Advancement of Research into Supportive, Palliative and End of Life Care, School of Health Sciences, University of Nottingham, Nottingham.; Primary Care Unit, Department of Public Health and Primary Care, University of Cambridge, Cambridge.

**Keywords:** anticipatory prescribing, decision making, end of life care, general practitioners, home palliative care, qualitative methods

## Abstract

**Background:**

GPs have a central role in decisions about prescribing anticipatory medications to help control symptoms at the end of life. Little is known about GPs’ decision-making processes in prescribing anticipatory medications, how they discuss this with patients and families, or the subsequent use of prescribed drugs.

**Aim:**

To explore GPs’ decision-making processes in the prescribing and use of anticipatory medications for patients at the end of life.

**Design and setting:**

A qualitative interview study with GPs working in one English county.

**Method:**

Semi-structured interviews were conducted with a purposive sample of 13 GPs. Interview transcripts were analysed inductively using thematic analysis.

**Results:**

Three themes were constructed from the data: something we can do, getting the timing right, and delegating care while retaining responsibility. Anticipatory medications were a tangible intervention GPs felt they could offer patients approaching death (something we can do). The prescribing of anticipatory medications was recognised as a harbinger of death for patients and their families. Nevertheless, GPs preferred to discuss and prescribe anticipatory medications weeks before death was expected whenever possible (getting the timing right). After prescribing medications, GPs relied on nurses to assess when to administer drugs and keep them updated about their use (delegating care while retaining responsibility).

**Conclusion:**

GPs view anticipatory medications as key to symptom management for patients at the end of life. The drugs are often presented as a clinical recommendation to ensure patients and families accept the prescription. GPs need regular access to nurses and rely on their skills to administer drugs appropriately. Patients’ and families’ experiences of anticipatory medications, and their preferences for involvement in decision making, warrant urgent investigation.

## INTRODUCTION

Poor symptom management during the last days of life at home can cause considerable distress for patients, their families, and clinicians.^1^^–^^5^ In the UK, Australia, and New Zealand, the prescribing of injectable anticipatory medications is promoted to optimise symptom control in the last days of life in the community and prevent crisis hospital admissions.^6^^–^^10^ Anticipatory medications are prescribed and dispensed ahead of need to a named patient.^6^ These are kept in the home and are used by visiting doctors or nurses if the patient is unable to take oral medications and develops symptoms of pain, agitation, nausea and vomiting, or respiratory secretions when they are dying.^6^^,^^7^ The intervention is intended to ensure rapid access to medications, particularly out of hours when there can be added delays in sourcing medication.^11^^,^^12^ Box 1 briefly describes standard practice in prescribing anticipatory medications in the UK.

**Box 1.** Standard practice in prescribing anticipatory medications in the UK^5^^,^^7^^,^^11^^,^^19^^,^^20^

Injectable drugs are pre-emptively prescribed to be administered ‘if needed’ to manage pain, agitation, nausea and vomiting, and respiratory secretions in the last days of life The drugs and doses prescribed vary depending on anticipated clinical need and local practice guidance, and may include sublingual lorazepam A box or bag containing the medications, needles and syringes, patient information sheet, and signed medication administration authorisation chart detailing doses to be given are kept in the patient’s home Visiting nurses or doctors make a clinical assessment before deciding whether to administer any medications

Previous research into anticipatory prescribing practice has primarily focused on the views and experiences of nurses.^5^^,^^10^^,^^11^^,^^13^^,^^14^ Nurses often report that they initiate anticipatory prescribing, requesting GPs prescribe the drugs weeks before likely need.^5^^,^^10^^,^^11^^,^^13^^–^^15^ Only two studies to date have investigated GPs’ views in any detail.^13^^,^^16^ GPs report prescribing drugs from a few days to short weeks before likely need, depending on their personal clinical preferences and the unpredictability of the patient’s illness.^16^

Nurses consistently report some GPs to be reluctant to prescribe anticipatory medications.^10^^,^^13^^,^^15^^,^^17^ Prescribing drugs ahead of need raises safety concerns for GPs.^13^^,^^16^^,^^18^ The GP remains accountable for the drugs they have prescribed, including strong opioids, which may be in the home for weeks. Once prescribed, permission has been granted for nurses to use the drugs based on their clinical assessment that the person is dying and has distressing symptoms.^10^^,^^11^^,^^19^ Drugs are open to misuse if there is a history of drug abuse in the home or they are given in inappropriate doses by visiting clinicians.^16^

Nurses and GPs generally perceive that ready access to anticipatory medications provides patients and families with reassurance.^5^^,^^11^^,^^20^ Community nurses report tailoring conversations about drugs depending on their own confidence and their perception of patients’ and family members’ willingness to talk about end-of-life care.^5^ What information GPs discuss with patients and families about anticipatory medications has not been investigated to date. This study explores GPs’ decision-making processes in the prescribing and use of anticipatory medications for patients at the end of life.

**Table:** How this fits in

The prescribing of anticipatory medications to provide symptom relief in last days of life care is recommended practice in the UK, Australia, and New Zealand. GPs have a central role in the prescribing of anticipatory medications, but little is known about their decision-making processes and how they discuss these with patients and families. This study found that GPs are keen to prescribe drugs weeks ahead of death even if they are unlikely to be needed. GPs often recall framing anticipatory medications as their clinical recommendation to ensure that the prescription is accepted by patients and their families.

## METHOD

### Design

This interpretive descriptive study^21^^,^^22^ was conducted in an English county with a mixture of urban and rural communities. Interviews were undertaken with 13 GPs practising in 13 different GP surgeries, about their views and experiences of decision making about end-of-life anticipatory medications. Potential participants were identified through publicly accessible information on GP practice websites. Purposive sampling sought GPs with a wide range of perspectives and experience, including geographical location and out-of-hours work.

Thirty-two potential participants were approached by letter inviting them to participate, with the study information sheet and reply form that requested information concerning the number of years worked as a GP and whether they had a particular interest in palliative care. Sixteen GPs replied to express interest in taking part.

### Data collection

Interviews were semi-structured, in depth, and conducted between June and December 2017 by one researcher, who is a clinical academic and community palliative care nurse with experience of conducting qualitative research. Participants took part in one-off audio recorded interviews that lasted 26–48 minutes. An interview guide explored participants’ perceived role in end-of-life care, their decision making about anticipatory medications in recent patient cases, and associated conversations with patients, their families, and other healthcare professionals. The interview guide was continually adapted in response to early data collection and analysis (see Supplementary data 1 for details).^23^

Twelve interviews were conducted face-to-face at the GP participants’ place of work or home address, and one was conducted by telephone. No new themes were identified after 11 interviews. However, two further interviews were undertaken to recruit participants with characteristics of interest and ensure a range of rich insights.^23^^,^^24^

### Data analysis

Interview recordings were professionally transcribed verbatim and checked by the interviewer for accuracy. Data were interpreted inductively using Braun and Clarke’s six phases of thematic analysis:^23^ data familiarisation; generating initial codes; constructing themes; reviewing potential themes; defining and naming themes; and producing the report. Transcripts were coded by hand and then using NVivo (version 11) by the interviewer.

To help reflexivity and rigour in interpreting the data,^25^^,^^26^ a public contributor with some experience in thematic analysis but no clinical training, independently coded the first three transcripts: and then compared and reflected on the early coding decisions together with the interviewer. A second researcher also read six transcripts and provided new insights throughout the coding process. These iterative steps informed the interviewer’s interpretative analysis.^23^^,^^27^^–^^29^ Data were synthesised to understand patterns and differences in GPs’ accounts.

## RESULTS

The sample comprised 10 GP principals (mean 16 years working as GP, range 3–29 years) and three salaried GPs (mean 5 years working as GP, range 2–10 years); five GPs also worked as out-of-hours doctors. Eight GPs were male and five female; five worked in urban practices, eight in rural settings. Four worked full-time and nine part-time, and five described themselves as having an interest in palliative care.

Three interconnected themes were constructed from the data (something we can do, getting the timing right, and delegating care while retaining responsibility), and are set out below. Pseudonyms are used throughout.

### Something we can do

Anticipatory medications represented a tangible intervention that GPs felt they could offer for patients approaching death when more active medical options became inappropriate: ‘something we can do’. All the GPs interviewed highlighted that it was essential to have the drugs in place as an insurance plan that could be used to provide end-of-life symptom relief if needed:

‘There’s no crystal ball and it’s better to have them in place than face some sort of crisis.’

(Dr Brown, GP)

Participants recalled finding it hard to accurately predict some patients’ likely death and symptom control needs, especially in fluctuating terminal illnesses such as advanced dementia or multiple illnesses and frailty. They generally prescribed drugs while patients were relatively stable, as it helped them manage the uncertainty. In some recalled cases, prescribed drugs remained in the home for months or went unused. This was not considered to be problematic, with potential risks perceived to be outweighed by the benefits of giving reassurance to patients, families, and clinicians:

‘We’ve certainly had a few people we’ve prescribed them so early they’ve gone out of date, which is kind of a bit silly, but actually if it’s giving them an extra bit of insurance along the way then I guess that’s okay.’

(Dr Smith, GP)

GPs wanted to put drugs in place to prevent potential problems for patients, families, and their colleagues out of hours. However, the five participants working in out-of-hours periods were not reliant on anticipatory medications being in place. They all had other strategies to get medication quickly when needed, including carrying limited supplies of drugs with them or collecting medication ahead of visits if they felt the patient was likely to need them:

‘We carry diamorphine and midazolam in a little safe in the car … If you haven’t left the base yet, you can anticipate and take it.’

(Dr Cook, GP and out-of-hours doctor)

Participants were working in, and promoted, a culture where it was desirable to plan for an expected death and prescribe drugs well ahead of time:

*‘One or two of the partners said, “I think it’s a problem thinking when to do it”. I said “Why? Why can’t you just leave it* [anticipatory medications] *gathering dust ... why can’t you do it early?”.’*

(Dr Taylor, GP)

Anticipatory medications were also used as a sign to alert other visiting clinicians to the terminal nature of the patient’s condition. Electronic records were not always shared between services. Consequently, clinicians unfamiliar with the patient’s situation had limited information on which to base their assessments. Having the drugs in the home sent a clear sign that the focus of care should be on providing end-of-life symptom control. Their presence also enabled doctors who did not know the patient to make remote care decisions:

‘I think in one way it makes life easier. Decisions can be made over the phone, then, with the district nurses. It makes it clear to everyone what’s going on, which I think is useful.’

(Dr Riplin, GP and out-of-hours doctor)

### Getting the timing right

The prescribing of anticipatory medications was recognised as a ‘harbinger of death’ for patients and their families: conversations needed handling with sensitivity and skill. Participants described some patients and families as pragmatic and willing to have the drugs in place, whereas others viewed their introduction as an unwelcome sign of approaching death:

‘I think some patients find it reassuring, other patients I think find it about as reassuring as seeing a coffin propped up in the corner of the room. It’s about being sensitive to the individual patients and their needs and their wants as well.’

(Dr French, GP)

Despite being aware of their symbolic significance, 12 participants recalled prescribing anticipatory drugs weeks before expected death. In contrast, one GP reported waiting until days before expected death and prescribed drugs when patients were unable to consistently swallow oral medication. Several GPs recalled cases where they had made the decision to prescribe drugs when patients were symptom-free but their care had overtly changed from active treatment to end-of-life care. These events stimulated GP decision making and made it easier to bring up the subject of anticipatory medications in the context of planning for end-of-life care:

‘I tend to do it [prescribe drugs] at the same time as we agree that we’re not going to resuscitate or admit someone to hospital.’

(Dr Aplin, GP)

GPs reported being receptive to nurses proactively requesting that they consider prescribing drugs. They also commented that it had become increasingly uncommon for community nurses to request drugs because community nursing services were so overstretched. All but one of the participants recalled that they would see the patient in person to judge for themselves if it was appropriate and acceptable to prescribe the drugs. One GP recalled there were occasions when they had prescribed drugs remotely at the request of nurses who they knew and trusted to have prescribing discussions with patients and families. Where patients were in nursing homes, the GPs relied on experienced nurses to prompt them to consider prescribing drugs:

*‘It’s easier when I’m in the nursing home with experienced nurses that are with a patient all the time. We can have a discussion, and then we decide, “No, we’ll do it* [prescribe anticipatory medications] *after the weekend, she’ll be okay, it doesn’t look like it’s imminent,” or, “Yes, let’s get everything ready before the weekend”.’*

(Dr Cox, GP)

GPs reported that they typically assumed responsibility for initiating anticipatory prescribing conversations with patients and families. They described their discussions with patients and families about anticipatory medications being incorporated in end-of-life advanced care planning conversations exploring patient and family understanding of the prognosis, preferred place of care, and death, and do not attempt cardiopulmonary resuscitation decisions. Just how commonly GPs had these conversations was unclear because three participants struggled to recall a recent end-of-life care case. Most GPs were keen to get what were seen as potentially difficult end-of-life conversations out of the way early so that plans were in place. Then patients, families, and participants did not have to worry about having distressing conversations in the future:

‘I like to have those conversations early. To get them out of the way sounds like I’m trying to avoid them, I think get them out of the way for their benefit so that they don’t have to, they can, there’s a lot to sort out, “let’s get it all sorted out and then enjoy the last time you have”.’

(Dr Matthews, GP and out-of-hours doctor)

GPs reported framing the idea of anticipatory medications in a way that matched their perceptions of the patient’s willingness to openly discuss death and dying. If patients and families voiced worries about dying in pain or distress, GPs described going into detail about what symptoms could occur and what anticipatory medications would do in specific situations. Conversely, if patients and families were perceived as being reluctant to consider what might happen during the dying process, GPs described providing minimal information about drugs and their role in care:

‘My experiences of talking to them, they vary as to what the patients seem to want to know … I just sort of try and explain that it’s there as a sort of mini pharmacy for people who are qualified to be able to access and sort of improve things without having to sort of go through the process of trying to get hold of the chemist.’

(Dr Taylor, GP)

Despite often recalling that patients and families appreciated having anticipatory medications prescribed, it was evident that participants often expected patients and families to be ambivalent about having them issued. Ten GPs described presenting anticipatory medications as a clinical recommendation, while giving patients or their families the opportunity to opt out of having them. This allowed GPs to present an illusion of supporting patient choice when they were using varying tactics of persuasion. For example, Dr Baker recalled using their position of authority to convince patients and families to have the drugs in the home when they judged this was in their best interests:

‘I’m sometimes a bit more paternalistic than I normally am. I would sometimes say “well actually these, this is the appropriate time and this needs to be something that we do”. So, I will sometimes slightly force that discussion and then take time to explain why but I would sometimes, kind of use the doctor card.’

(Dr Baker, GP and out-of-hours doctor)

### Delegating care while retaining responsibility

GPs were aware that they remained responsible for anticipatory medications once prescribed, but had little knowledge or control over when they were used:

‘I’ve prescribed it in a way to be used and I’d expect them to use it … I don’t think every time it’s used I need to know.’

(Dr Baker, GP and out-of-hours doctor)

Delegating to unknown nurses or doctors the responsibility for assessing when to administer drugs caused concern at times:

‘There are some issues about writing up potentially life-terminating drugs if used in an inappropriate way … to be used at the discretion of a third party, with no connection between the third party who initiates them and the prescriber.’

(Dr French, GP)

GPs were more concerned that drugs might not be used when they were needed. They were frustrated when nurses did not recognise dying and failed to administer drugs to relieve symptoms:

*‘That was not a good death … she became acutely unwell and breathless quite suddenly. The son was very distressed because he was with her and didn’t understand what was happening, wasn’t able to be reassured by the* [nursing home nurses] *that she was dying ... They didn’t give her anything, they just called an ambulance.’*

(Dr Jones, GP)

GPs were reluctant to leave controlled drugs in the home if there was a history of drug misuse in the family. If drugs were left in the home for extended periods, GPs would often rely on nurses to monitor for potential risks and provide feedback on whether the prescriptions remained appropriate:

‘So, it’s just not leaving boxes and ampoules of medications, but doing it at a time when the support network has been built in … and assessing safety as well.’

(Dr Lewis, GP)

Having easy access to nurses was perceived to be crucial in facilitating good end-of-life care and the appropriate use of anticipatory medications. All participants highlighted the importance of being able to have telephone or face-to-face conversations with nurses to get to know their skills and abilities, and to keep updated on patients’ end-of-life care. Structural changes to the community nursing service meant that nurses were no longer based in the same building as GPs and communications went via a centralised contact centre. Telephone and electronic messages had replaced face-to-face and informal routes of communication, which had previously facilitated close working relationships. GPs were frustrated with this new arrangement and believed it had a negative impact on interprofessional relationships, communication, and patient care:

*‘It’s potentially quite disjointed from the feedback we’ve had from patients … We moved from the district nurses being in the practice* [who] *we could see every day … to now calling a number and not knowing who we were going to speak to.’*

(Dr Pegg, GP and out-of-hours doctor)

With less personal contact with community nurses, the GPs had become increasingly reliant on shared electronic patient records to review nursing care remotely. Participants reported being keen to also maintain regular interdisciplinary team meetings to discuss patients’ end-of-life care needs. They described increasing difficulty in ensuring that community nurses attended these meetings, commenting that patient care was suffering as a result. Despite these issues, the role of community nurses in end-of-life care was highly valued by the GPs who recognised that they prioritised these patients and worked hard to meet their needs:

‘I know that there’s a lot of criticism about the lack in district nursing support in general … but in terms of end-of-life I think they are really good because they are there whenever I have people.’

(Dr Lewis, GP)

## DISCUSSION

### Summary

This study found that GPs prefer to prescribe anticipatory medications weeks ahead of likely need whenever possible. They recall framing information about the drugs and their uses in ways that ensure that patients and families are willing to have them in the home. After prescribing anticipatory medicines, GPs rely on nurses to judge when to administer drugs and to keep them updated on patients’ end-of-life care.

### Strengths and limitations

This study offers new and detailed insights into GPs’ perceptions of their end-of-life care prescribing and decision making, from in-depth interviews with a diverse group of GPs. Although limited to one English county, the mixture of urban and rural practice settings offers valuable insights that are transferable across the UK.

The interviews reflect GPs’ accounts of their own practice, rather than detailing actual practice. Interviewees can focus on notable cases or present versions of events, which fit within acceptable professional norms.^30^

The interviewer’s previous clinical role as a community palliative care nurse helped with understanding working cultures and aided participant–researcher rapport.^28^ To ensure data were not interpreted purely through the interviewer’s personal clinical lens, a public contributor and second researcher contributed to data analysis.^23^^,^^28^^,^^31^ Key decision points in the analysis were also debated with two researchers to help achieve a comprehensive and reflexive analysis.^23^^,^^29^

### Comparison with existing literature

GPs’ accounts of actively leading the decisions to prescribe anticipatory medications in this study challenge the published perspective that it is nurses who routinely decide when drugs should be issued.^5^^,^^10^^,^^11^^,^^13^^,^^15^ GPs have previously been presented as being cautious of prescribing anticipatory medications unless they are likely to be needed within days or short weeks.^16^ In contrast, the current study found that most GPs reported they commonly prescribe drugs weeks ahead of expected death. Participating GPs considered it important to have drugs available even if they were unlikely to be needed, reflecting national guidance,^6^^,^^7^ and the preferences of nurses.^5^^,^^10^^,^^11^^,^^14^ Accounts of prescribing drugs weeks or months ahead of death also reflected the prognostic challenges in end-of-life care for patients increasingly dying of non-cancer conditions such as dementia, ischaemic heart disease, and multimorbidity in old age,^16^^,^^32^ for whom illness trajectories are commonly less predictable and the dying phase protracted.^33^^,^^34^

Leaving anticipatory medications in the home for extended periods of time raises safety concerns.^16^ Patient safety may be compromised if drugs are administered without a prior skilled clinical assessment to rule out any reversible causes, diagnose dying, and check that the prescription remains appropriate.^11^^,^^12^^,^^18^ A 2019 analysis of patient safety incident reports found a recurring lack of knowledge or skills in using anticipatory medications among out-of-hours nursing and medical staff.^35^

The current study is the first to identify that anticipatory medications are used as a sign to alert other visiting clinicians to the terminal nature of the patient’s condition. Having the drugs in place enabled doctors unfamiliar with the patient to make care decisions without visiting. Regular reviews by skilled clinicians who know the patient and their situation are a central component in high-quality end-of-life care.^36^^–^^38^ In a climate where there are increasing demands on overstretched GP and community nursing services,^37^^,^^39^^,^^40^ there is a danger that anticipatory medications may be used to substitute regular clinical reviews by familiar GPs and community nurses. These risks are exacerbated during the COVID-19 pandemic as medical reviews by telephone or video are becoming the norm.^41^

Mirroring the findings of a study of community nurses’ experiences of anticipatory medication prescribing conversations,^5^ participating GPs reported that patients and families could view the drugs as an unwelcome reminder of approaching death. Community nurses reported that some patients were reluctant to have drugs prescribed as a result.^5^ Patients and families may be more inclined to accept GPs’ recommendations because of a greater power imbalance in the doctor–patient relationship.^42^^,^^43^ GPs in the current study recalled framing anticipatory medications as a clinical recommendation, using persuasive language or their authority to ensure the prescription was accepted. Similar techniques of persuasion have been reported in studies investigating how clinicians’ selectively present information about end-of-life care interventions if patients are hesitant to accept care or reluctant to have detailed end-of-life discussions.^44^^–^^46^

In the current study, once GP participants had prescribed anticipatory medications they left nurses to manage end-of-life care and decide if and when to administer the drugs. This matches with other researchers’ findings that GPs often assume the role of ‘medical consultant’, delegating day-to-day care to community nurses, and relying on them to access GP input only if needed.^47^^,^^48^ Previous research has found that established relationships of trust between GPs and nurses, respect for each other’s expertise, and ease of access to each other, is important in ensuring that anticipatory medications are prescribed and used appropriately.^5^^,^^13^ The current study found that even when GPs have infrequent contact with community nurses and have limited knowledge of their skills, they still delegated care based on historical relationships of trust. However, relationships of trust are becoming increasingly difficult to establish and maintain as organisational changes have resulted in more distant and fragmented communication.^13^^,^^49^^–^^51^ This changing relationship could have negative impacts on established practices of anticipatory prescribing,^5^^,^^13^ and on GPs’ confidence in delegating day-to-day end-of-life care to community nurses.^47^^–^^49^

### Implications for research and practice

Anticipatory medications are viewed as a key component in community end-of-life practice. It has become culturally acceptable for GPs to prescribe anticipatory medications weeks or even months before death is expected, in part to address the uncertainties of when the dying phase will start.^5^^,^^6^^,^^34^ There is a risk that such advance anticipatory prescribing might hinder the recognition and treatment of reversible causes of symptoms, especially when decisions about care are made remotely, and in the absence of skilled clinical face-to-face GP or specialist reviews of the patient.^18^^,^^41^ Accounts of using the presence of anticipatory medications to guide care decisions in lieu of adequate sharing of patient electronic care plans between services raises questions about patient safety. The safety of prescribing drugs weeks ahead of possible need warrants further research.^12^

Robust integrated systems are needed across primary and community care services to ensure that drugs and doses are reviewed regularly and are only administered when clinically appropriate.^6^^,^^18^ In order to feel comfortable delegating care once they have prescribed anticipatory medications, GPs need to have regular contact with nurses and trust in their skills to administer drugs when clinically indicated. Informal routes of communication and regular interdisciplinary team meetings between GPs and nurses are also vital in maintaining strong working relationships and coordinating patient-centred end-of-life care.^5^^,^^13^^,^^49^^,^^50^

The way anticipatory medications are framed in GPs’ accounts of their prescribing conversations raises questions about how well informed patients and their families are about the drugs, potential side-effects, and their role in end-of-life care at home. Relying on clinicians’ assumptions that anticipatory medications provide reassurance risks misunderstanding patients’ and families’ concerns and wishes.^5^^,^^52^^,^^53^^,^^54^ No study to date has investigated patients’ views and experiences of anticipatory medications, and their preference for involvement in decision making.^12^ The authors have a study currently underway addressing this knowledge gap.
